# Acid/Alkali-Resistant Modified MOF-74 Grafted with Polyether Demulsifier for Oil-in-Water Emulsions Under Ambient Conditions

**DOI:** 10.3390/polym17172386

**Published:** 2025-08-31

**Authors:** Bingyu Wang, Wei Guo, Ying Deng, Wenbin Jiao, Linzhu Du, Junhui Yue, Bo Zhang

**Affiliations:** 1 National Engineering Laboratory for Advanced Municipal Wastewater Treatment and Reuse Technology, Beijing University of Technology, Beijing 100124, China; 2 Chinese Research Academy of Environmental Sciences (CRAES), Beijing 100012, China

**Keywords:** MOF-74, nonionic polyether, silane coupling agent, oil-in-water emulsion, interfacial activity, demulsification mechanism

## Abstract

The effective and rapid separation of oil–water emulsions at room temperature, particularly under harsh environmental conditions like acid–base fluctuations, high salinity, and the coexistence of surfactants, remains a significant challenge in oily wastewater treatment. To address this, a novel amphiphilic demulsifier, MOF-74@SiO_2_-GPTMS grafted ANP (MSG-ANP), was synthesized by first modifying MOF-74@SiO_2_ (MS) with γ-glycidoxypropyltrimethoxysilane (GPTMS) to create epoxy-functionalized MSG particles, followed by grafting the non-ionic polyether C_12_–C_14_ aliphatic polyethylene oxide polyoxypropylene (ANP) onto MSG. Bottle tests demonstrated that MSG-ANP achieved a high demulsification efficiency of 93% within 15 min for oil-in-water emulsions at room temperature. It exhibited excellent environmental tolerance, maintaining efficiencies of 89% at pH 3.0, 82% at pH 11.0, and 95% under high salinity (50,000 mg/L, pH 6.8). Furthermore, MSG-ANP effectively treated surfactant-stabilized emulsions, exceeding 96% efficiency against both cetyltrimethylammonium bromide and sodium dodecyl sulfate after 30 min, outperforming commercial demulsifiers SP-169 and AR-331 by factors of 1.2 and 1.6, respectively. This superior performance stems from synergistic hydrogen bonding (via hydroxyl, ether, ester, Fe-O, and Si-O groups) destabilizing the interfacial film and electrostatic neutralization of coalescing charged droplets. Consequently, MSG-ANP presents a promising solution for rapid, room-temperature demulsification across a wide pH range and under high-salinity conditions.

## 1. Introduction

Oil-in-water (O/W) emulsions are widely found in a variety of industrial processes, including petrochemical, metallurgical, pharmaceutical, and food and beverage industries [1]. The formation of these emulsions is mainly due to the interfacial action of some surfactants in oily wastewater that reduces the likelihood of oil droplet aggregation, resulting in the stable dispersion of fine oil droplets (especially for oil droplets less than 20 µm) in the continuous aqueous phase [2,3]. Moreover, the complex water qualities associated with these industrial wastewaters, such as broad pH ranges and high salinity, not only further enhance emulsion stability by altering the surface charge or compressing the interfacial bilayer but also increase the difficulty in separating emulsified oil or water [4,5]. This challenge is compounded by environmental factors; as demonstrated in colloidal systems research, external conditions (e.g., temperature) significantly reduce separation efficiency [6]. Generally, these untreated emulsions are undesirable and even harmful to environment ecosystems and human health in many situations [1]. In addition, the presence of emulsions can lead to pipeline blockage and equipment corrosion during the storage and transportation of oil or oily wastewater [7]. Therefore, the development and application of efficient oil–water separation technologies are of paramount importance to mitigate the environmental and health impacts associated with O/W emulsion wastewaters.

At present, the application of oil–water separation technologies mainly includes physical, biological, and chemical methods [8,9]. Among them, chemical demulsification is currently considered to be the most efficient and convenient [10]. Chemical demulsifiers such as polyether-types, quaternary ammonium salts, and phosphate esters have amphiphilic structures, including hydrophobic and hydrophilic groups, which can contact with the oil–water interface, reduce the interfacial tension, destroy the interfacial membrane, and promote the separation of oil and water from O/W emulsions [11]. Recent studies on interfacial adsorption behavior further confirm that surface interactions play a critical role in destabilizing emulsions [12]. Additionally, chemical demulsification is simpler and more flexible in application, and the demulsification process can be controlled by the selection and dosage of the demulsifier [3]. However, this approach also faces some challenges, including the need to increase the medium reaction temperature, prolong the reaction time, and overcome extreme conditions such as strong acids or bases and high salts with low demulsification efficiency [13]. Therefore, how to improve the adaptability of demulsifiers to various emulsion properties and environmental conditions has been the focus of chemical demulsification research recently [14].

With tunable surface properties and high interfacial activity, nanoparticle demulsifiers have gradually become ideal chemical demulsifier candidates to cope with complex emulsions and harsh environments in oily wastewater [15]. A variety of nanoparticles, such as silica [16], carbon nanotubes [17], magnetic (Fe_3_O_4_) nanoparticles [18], and metal–organic frameworks (MOFs) [19], have been used to construct nanoparticle demulsifiers. Among these nanomaterials, MOFs are crystalline porous coordination materials formed by metal ions connected through multitopic organic ligands with a polar node and non-polar skeleton [20]. This structure endows MOFs with amphiphilic microdomains, high porosity, and a large surface area, as well as facile preparation and easy tunabiltiy, which gives it great potential for oil–water separation and emulsion demulsification [21,22]. For example, NCFM-0.3, derived from Ni-MOF nanosheets modified with carbon fiber composite membranes, exhibits the highest oil–water separation flux of 7279 L m^−2^ h^−1^ for the saline oil-in-water emulsions [23]. Due to the increased roughness and low surface energy of the cotton balls with Sm-MOF, the modified cotton balls have excellent superhydrophobicity (high water contact angle of 150.75 ± 1.1°) and oil–water separation efficiency (>95%) [24]. However, most of the current superhydrophobic/superlipophilic MOF materials have only made progress in immiscible oil/water separation, and most of them are focused on applications modified with other materials as separation membranes [25,26]. The potential of MOF materials to be used directly as demulsifiers remains to be exploited, especially in the treatment of miscible oil/water emulsions with small droplets in harsh chemical environments.

Among various MOFs, MOF-74 has a remarkable advantage in oil–water separation, which is mainly attributed to its high surface energy and large adsorption capacity to effectively reduce the interfacial tension between oil and water [27,28]. However, due to the limitations of chemical dispersion and stability of nanoparticles, it is difficult to effectively improve the emulsion breaking efficiency in harsh environments [29]. This may also have constrained research into the direct use of MOF materials as demulsifiers. To improve the intrinsic properties of nanoparticles and address the challenges of their aggregation and stability in harsh environments, strategic surface functionalization techniques were employed [3]. Silane coupling agents can covalently modify MOFs and significantly increase the surface functional group density. Specifically, γ-glycidoxypropyltrimethoxysilane (GPTMS) has a dual reactive site that forms strong Si-O-Si bonds with the silica layer while exposing epoxy groups for subsequent reactions. This modification greatly improves interfacial activity and particle stability under extreme pH and salinity conditions [30]. Most importantly, the terminal epoxy groups of GPTMS can act as an anchor for polymer grafting, thus enabling the ability to break emulsion at room temperature, which is a critical step towards practical applications. In addition, grafting an oxygen-rich nonionic polymer, C_12_-C_14_ aliphatic alcohol polyoxypropylene (ANP), onto the epoxy-functionalized surface of the nanoparticles can further optimize the material’s wettability and may enhance interfacial disruption at room temperature [31]. The efficacy of grafted ANP lies in its ability to hydrogen-bond with emulsion components while regulating the amphiphilic equilibrium of the nanoparticles, thereby promoting droplet coalescence of the emulsion under mild conditions [32,33]. Therefore, with the above sequential modification strategy of silica stabilization, GPTMS coupling and ANP grafting can overcome the shortcomings of MOF nanoparticles, thereby enabling MOF nanoparticles as an emulsion-breaking agent to rapidly break emulsions at room temperature and in extreme chemical environments. However, there have been no reports on the functional modification of nanomaterials such as MOF-74 and the grafting of non-ionic polyether ANP as a demulsifier.

Thus, we designed a novel demulsifier (MSG-ANP) by grafting the nonionic polyether ANP onto GPTMS-functionalized MOF-74@SiO_2_ (MSG). This strategy combines three advantages: (i) the high interfacial activity of open metal sites of MOF-74; (ii) enhanced acid and alkali stability imparted by GPTMS-derived siloxane networks; and (iii) flexible hydrogen-bonding sites provided by ANP chains for rapid interfacial film disruption. The objectives and contents of this work include the following: (i) synthesizing MSG-ANP via a scalable silane–polyether grafting approach and optimizing its amphiphilicity (by adjusting the MSG:ANP mass ratio); (ii) systematically evaluating its performance under various extreme conditions at room temperature (pH 3–11, salinity ≤ 50,000 mg/L, emulsions of diverse surfactant types), and comparing it with commercial demulsifiers SP-169 and AR-331; (iii) investigating the morphological characteristics and demulsification mechanism of the synthesized demulsifier by scanning electron microscopy, high-resolution transmission electron microscopy coupled with energy-dispersive X-ray spectroscopy, Fourier-transform infrared spectroscopy, X-ray photoelectron spectroscopy, X-ray diffractometry, thermogravimetric analysis, zetasizer, contact angle meter, and interfacial tension measurements. These advances provide an effective implementation path for realizing MSG-ANP as an efficient treatment method for oily wastewater under extremely challenging practical conditions.

## 2. Materials and Methods

### 2.1. Materials

N,N-dimethylformamide (DMF), γ-glycidoxypropyltrimethoxysilane (GPTMS, 97%), C_12_-C_14_ aliphatic polyethylene oxide polyoxypropylene (ANP, 99%), sodium dodecyl sulfate (SLS, 98%), cetyltrimethylammonium bromide (CTAB, 99%), Tween 80, toluene (99.5%), n-hexane (97%), and anhydrous ethanol (water ≤ 0.3%) were supplied from Shanghai Macklin Biochemical Co., Ltd. (Shanghai, China). Cobalt nitrate hexahydrate (Co (NO_3_)_2_·6H_2_O, 99%) and Ferrous chloride tetrahydrate (FeCl_2_·4H_2_O, 99.95%) were obtained from Shanghai Aladdin Biochemical Technology Co., Ltd. (Shanghai, China). 2,5-Dihydroxyterephthalic acid (2,5-DHTPA) was ordered from Shanghai Acmec Biochemical Technology Co., Ltd. (Shanghai, China), while tetraethyl orthosilicate (TEOS, 98%) was purchased from Tokyo Chemical Industry Co., Ltd. (Tokyo, Japan). Diesel was provided by Dagang oilfield in Tianjin, China. All chemical reagents were of analytical grade and were utilized without further purification.

### 2.2. Demulsifier Preparation

**Figure 1:**
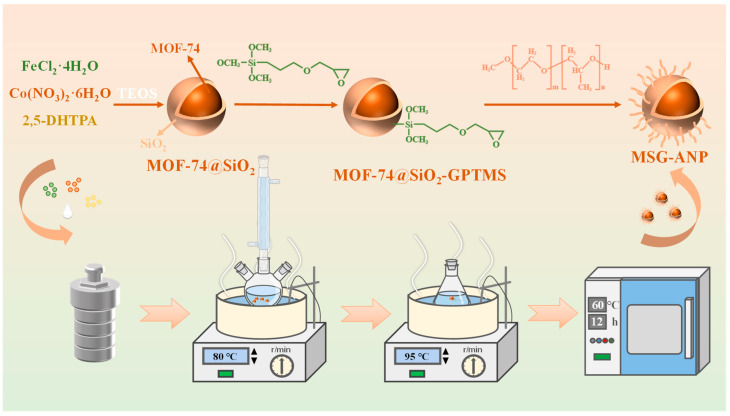
Synthesis of MSG-ANP.

The synthesis route for the demulsifier is illustrated in Figure 1. Firstly, MOF-74@SiO_2_ (MS) was synthesized according to the reported method [34]. A quantity of 3 mmol (596 mg) of FeCl_2_·4H_2_O, 1.5 mmol (436 mg) of Co (NO_3_)_2_·6H_2_O, 1.5 mmol (297 mg) of 2,5-DHTPA, and 0.25 mL of TEOS were dissolved in 65 mL of a mixed solvent composed of DMF, ethanol, and ultrapure water in a ratio of 10:2:1. Following this, an additional 0.25 mL of TEOS was introduced to the solution, which was stirred for 2 h. The resulting mixture was then transferred to a Teflon-lined autoclave and subjected to continuous reaction at 120 °C for 24 h. After the reaction, the precipitate was collected through centrifugation at 9000 rpm for 10 min, washed multiple times with DMF, ultrapure water, and ethanol, and subsequently dried under vacuum at 80 °C for 6 h to obtain MS.

Secondly, MOF-74@SiO_2_-GPTMS (MSG) was synthesized according to the reported method [18]. MS (1200 mg) was added to toluene (240 mL) and ultrasonically dispersed at 100 W for 30 min. Subsequently, the ultrasonicated mixture was transferred to a three-neck round-bottom flask, and GPTMS (8 mL) was added. The mixture was purged under a nitrogen (N_2_) atmosphere 2–3 times at room temperature. Thereafter, the mixture was stirred continuously for 8 h at 80 °C under N_2_ atmosphere. Finally, the solid product was separated from the solvent by centrifugation at 8000 rpm for 6 min, washed several times with anhydrous ethanol, and dried for 12 h in a vacuum oven at 60 °C to obtain MSG.

Finally, MSG-ANP was obtained according to the reported method [35]. Different mass ratios (8:1, 4:1, 2:1, 1:1, and 1:2) of MSG to ANP (see Table S1 for details) were mixed with toluene (85 mL) in a 150 mL conical flask and ultrasonically treated at 100 W for 20 min at 25 °C. The solid phase was then separated from the mixture by centrifugation at 9000 rpm for 6 min and washed three times with anhydrous ethanol. Finally, the solid phase material was dried in a vacuum oven at 60 °C for 12 h to obtain MSG-ANP.

### 2.3. Preparation of O/W Emulsion and Demulsification Test

Three distinct surfactant-stabilized oil-in-water (O/W) emulsions were prepared: CTAB (cationic)-emulsion, SLS (anionic)-emulsion, and Tween 80 (nonionic)-emulsion, each containing 1 g/L diesel oil. Specifically, 0.50 g diesel was mixed with 500 mL deionized water containing 20 mg/L surfactant (CTAB, SLS, or Tween 80) at ambient temperature. The mixtures were ultrasonicated at 100 W for 1 h to ensure uniform dispersion. Remarkably, the prepared emulsions exhibited stability at room temperature for two weeks, as visually confirmed by the unchanged droplet morphology in Figure S1.

The bottle test method was employed to evaluate the demulsification efficiency of MSG-ANP. The pH of emulsions was adjusted to 3–11 by adding 1 M HCl or 1 M NaOH, while salinity was modified to 0–50,000 mg/L through NaCl addition. A certain amount of demulsifier was added to 10.0 mL of emulsion in a colorimeter tube and shaken vigorously at 200 rpm for 2 min using a mechanical shaker. Subsequently, a static reaction without stirring was carried out at 25 °C for a specified duration, and the demulsifying process was monitored. The oil content before and after demulsification was quantified using a UV-Vis spectrophotometer (TU-1810, Beijing Purkinje General Instrument Co., Ltd., China, Beijing, China) at a wavelength of 256 nm to determine its content according to the previous study [36]. The demulsification efficiency was calculated using Equation (1):

(1) $$\mathrm{DE}\;=\frac{C_{0}-C}{C}\times 100$$

where DE represents the demulsification efficiency (%), *C*_0_ is the oil content in the aqueous phase after demulsification (g/L), and *C* is the oil content in the prepared emulsion (g/L).

### 2.4. Characterization

The surface morphology of the synthesized demulsifier was characterized using a scanning electron microscope (SEM, S-4800, Hitachi, Tokyo, Japan). High-resolution transmission electron microscopy (HRTEM, Tecnai G2 F30, FEI, Hillsboro, OR, USA) coupled with energy-dispersive X-ray spectroscopy (EDS) was employed to examine the crystal structure and elemental distribution of the demulsifier. Fourier-transform infrared spectroscopy (FT-IR, Nicolet 6700, Thermo Fisher Scientific, Waltham, MA, USA) identified functional groups within the demulsifier, operating at 400–4000 cm^−1^ with 4 cm^−1^ resolution; each spectrum was averaged over 32 scans. The elemental composition of the demulsifier was analyzed via X-ray photoelectron spectroscopy (XPS, Escalab 250XI, Thermo Fisher Scientific, Waltham, MA, USA), using Al Kα as the excitation source, with the binding energy of the C 1s peak at 284.8 eV serving as the calibration reference. The crystal structure of the demulsifier was further investigated using an X-ray diffractometer (XRD, D8 ADVANCE, Bruker, Ettlingen, Germany) with a scanning rate of 3°/min across a 2θ range of 10–80°. Thermogravimetric analysis (TGA, TG 209 F3 Tarsus, NETZSCH, Selb, Germany) assessed the thermal stability of the demulsifier under N_2_ atmosphere, heating from 25 °C to 900 °C at 10 °C min^−1^. The wettability of the demulsifier at oil–water–solid interfaces was quantified via the sessile drop method using a contact angle meter (OCA50, Dataphysics, Filderstadt, Germany) at ambient temperature. The powder was first compressed at 10 MPa for 30 s to yield a smooth, cylindrical pellet approximately 1 mm thick by 13 mm in diameter. A droplet of ultrapure water (3.0 μL) was gently deposited onto the pellet surface using a micro-syringe; the reported contact angles are the average of at least three measurements taken at different positions after 10 s of equilibration. The dynamic interfacial tension (IFT) between diesel (oil phase) and demulsifier-containing aqueous phases was evaluated by the pendant drop method. Zeta potential and hydrodynamic diameter of demulsifier aggregates were measured by a zetasizer (Nano ZS, Malvern, UK) at ambient temperature. The sample (2 g L^−1^ MSG-ANP) was dispersed in deionized water, and the pH was individually adjusted to 3, 5, 7, 9, and 11 using 0.1 M HCl or 0.1 M NaOH prior to each determination. Finally, optical microscopy (Olympus, U-RFL-T, Tokyo, Japan) directly observed microstructural changes in emulsions before and after demulsification.

## 3. Results

### 3.1. Characterization of MSG-ANP

#### 3.1.1. Surface Morphology of MSG-ANP

**Figure 2:**
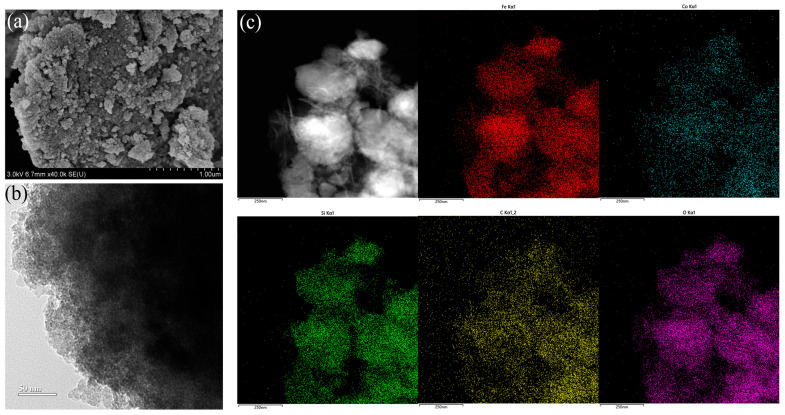
SEM image (**a**), TEM image (**b**), and HRTEM-EDS images (**c**) of MSG-ANP_3_(mass ratios of MSG to ANP: 2:1).

The morphology of MSG-ANP was analyzed using SEM and HRTEM. SEM images (Figure 2a) illustrated that MSG-ANP was characterized by an approximate spherical shape. Further analysis through HRTEM (Figure 2b) revealed the structural features of the core–shell of MSG-ANP with an average particle size of 58.5 nm (Figure S2). The elemental maps obtained by HRTEM-EDS confirmed the presence of Fe, Co, Si, C, and O, thus providing definitive evidence for the chemical composition of MSG-ANP (Figure 2c). Notably, the line-scan image of HRTEM (Figure S2) shows a low concentration distribution of Si in the central region of the particles and a higher concentration at the edges, which supports the formation of a Si core–shell structure around MOF-74.

#### 3.1.2. Comprehensive Chemical and Structural Characterization of MSG-ANP

**Figure 3:**
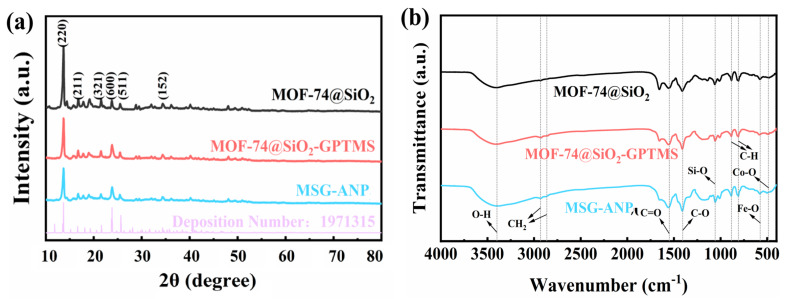
F XRD patterns (**a**) and FT-IR spectra (**b**) of MS, MSG, and MSG-ANP_3_ (mass ratios of MSG to ANP: 2:1).

XRD analysis further confirmed the crystal structure of MS (Figure 3a), revealing a diffraction pattern similar to that of MOF-74, as documented by the Cambridge Crystallographic Data Centre (CCDC), number 1971315. The diffraction peaks at 13.67°, 16.67°, 21.59°, 23.86°, 25.71°, and 34.35° correspond to the crystal planes (220), (211), (321), (600), (511), and (152) of MOF-74, respectively [37]. Notably, these diffraction peaks also appear in the XRD patterns of MSG and MSG-ANP, indicating that the crystal structure of MOF-74 is well preserved during the silica coating, modification with GPTMS, and the subsequent grafting of ANP.

The FT-IR analysis results are presented in Figure 3b. The infrared absorption peaks at 456 cm^−1^ and 526 cm^−1^ correspond to the stretching vibrations of Co-O and Fe-O, respectively [38]. The stretching vibration of Si-O is observed at 1056 cm^−1^ [34]. Additionally, the absorption peaks at 884 cm^−1^ and 810 cm^−1^ are attributed to the bending vibrations of the C-H bonds in the phenyl rings of the MOF-74 organic ligands [39]. The presence of these characteristic peaks provides spectroscopic evidence for the successful synthesis of MS [38]. The stretching vibrations of the C=O in the carbonyl group and the C-O in the ether bond are represented by the absorption peaks at 1550 cm^−1^ and 1407 cm^−1^, respectively [18]. The characteristic absorption peak at 3398 cm^−1^ is attributed to the stretching vibration of O-H in ANP, while the bending vibration peaks of -CH_2_ in ANP appear at 2931 cm^−1^ and 2864 cm^−1^, confirming the successful grafting of ANP onto MSG [35].

#### 3.1.3. Effect of Different Mass Ratios of MSG to ANP on MSG-ANP Properties

MSG-ANP with varying mass ratios of MSG to ANP were investigated in detail using XPS to obtain comprehensive surface chemical information. The XPS spectra presented in Figure S3 reveal that MSG-ANP, with different mass ratios of MSG to ANP, contains the elements Fe, Co, Si, C, and O. The high-resolution C 1s spectra shown in Figure S3a exhibit peaks at 284.8 eV, 286.3 eV, and 288.7 eV, which correspond to C-C, C-O-C, and O-C=O bonds, respectively, aligning with the results from FT-IR [40]. Additionally, the π-π* satellite peak observed at 292.4 eV is attributed to the presence of a benzene ring in the MOF-74 organic ligand [41]. In the Fe 2p spectra (Figure S3b), two distinct peaks at 711.2 eV and 724.0 eV are attributed to Fe^2+^ in Fe-O, while peaks at 714.7 eV and 727.0 eV are associated with Fe^3^⁺ [42]. The high-resolution Co 2p spectra reveal main peaks at 782.5 eV and 798.0 eV for Co 2p3/2 and Co 2p1/2, respectively (Figure S3c), with corresponding satellite peaks at 788.7 eV and 804.0 eV, indicating that cobalt predominantly exists in the Co^2⁺^ state [43]. As the mass ratio of MSG to ANP decreased, the intensity of the C 1s peaks increased, while the intensities of the Fe 2p and Co 2p peaks decreased, suggesting an increase in the content of ANP within the MSG-ANP composite. The observed chemical states and elemental compositions confirm the successful synthesis of MSG-ANP.

**Figure 4:**
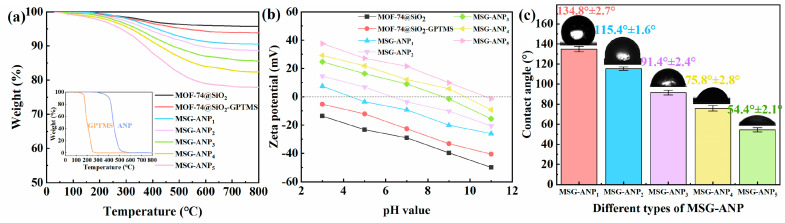
TGA curves of MS, MSG, and MSG-ANP with different mass ratios of MSG to ANP; inset are the TGA curves of GPTMS and ANP (**a**), zeta potential (**b**), and CA of MSG-ANP with different mass ratios of MSG to ANP (**c**).

The molar content of ANP on the demulsifier surface was quantified by TGA. As illustrated in Figure 4a, MS and MSG exhibited weight losses of 4.24% and 6.18% at 800 °C, respectively, primarily attributed to surface dehydration and dihydroxylation [44]. The complete decomposition of pure GPTMS and ANP at 800 °C (as shown in the inset of Figure 4a) indicates that MSG-ANP experiences significant weight loss in the temperature range of 200–800 °C, likely due to the decomposition of GPTMS and ANP on the surface of MS. The weight losses of MSG-ANP_1_ through MSG-ANP_5_ were recorded at 9.49%, 11.36%, 14.49%, 17.65%, and 22.09%, respectively, in the order of decreasing mass ratio of MSG to ANP. This trend demonstrates that the weight loss of MSG-ANP increases progressively with the amount of grafted ANP (Figure 4a). The molar contents of grafted ANP, calculated from the TGA results, were found to be 0.08, 0.12, 0.20, 0.27, and 0.37 mmol/g. These findings not only provide a quantitative basis for the molar content of ANP in MSG-ANP but also further corroborate the composition of the demulsifier.

Figure 4b illustrates the variations in zeta potential for MS, MSG, and MSG-ANP with varying mass ratios of MSG to ANP across different pH values. It was observed that the surface charge of MSG-ANP diminishes as the pH increases. This behavior can be explained by the differing interactions of -OH groups on the MSG-ANP surface under varying pH conditions. In acidic environments, these groups are prone to protonation, resulting in the formation of -OH2⁺, whereas in alkaline conditions, they deprotonate to become -O^−^ [45]. Furthermore, the zeta potential exhibited a gradual increase towards a positive value as the mass ratio of MSG to ANP decreased. This trend may facilitate the reduction of repulsive forces between emulsified droplets through electrostatic neutralization, thereby promoting demulsification [46]. These observations substantiate the role of the silane coupling agent and the grafting of ANP in altering the surface charge density of MS, further confirming that chemical modifications can modulate the charge density of the material from negative to positive. This phenomenon can be attributed to the nonionic character of ANP, which, despite lacking an inherent charge, has a hydrophobic group that adsorbs onto MSG. Simultaneously, its hydrophilic group interacts with the aqueous phase, thereby forming a thicker hydration layer. This layer acts as a spatial site barrier, effectively shielding the negative charge of the MSG particles [47]. A more detailed discussion on the correlation between zeta potential and demulsification performance will be provided in Section 3.2.

The water–solid–gas three-phase contact angle results for MSG-ANP samples with different mass ratios of MSG to ANP are presented in Figure 4c. As the mass ratio of MSG to ANP decreases, CA of MSG-ANP also decreases. This phenomenon can be attributed to the presence of lipophilic benzene rings, siloxane chains, and methyl groups in MSG, in contrast to the grafted ANP, which features hydrophilic ether bonds and hydroxyl groups [48]. By increasing the proportion of ANP in MSG-ANP, the wettability of the demulsifier surface can be effectively adjusted. The contact angle of 91.4° for MSG-ANP_3_ approaches the theoretical optimal value (90°) for amphiphilic materials, where balanced affinity to both oil and water phases maximizes adsorption at oil–water interfaces [49,50]. This near-neutral wettability reduces the energy barrier for demulsifier migration to the interface, facilitating displacement of surfactant films and coalescence of oil droplets [51].

### 3.2. Demulsification Performance of MSG-ANP Under Different Conditions

**Figure 5:**
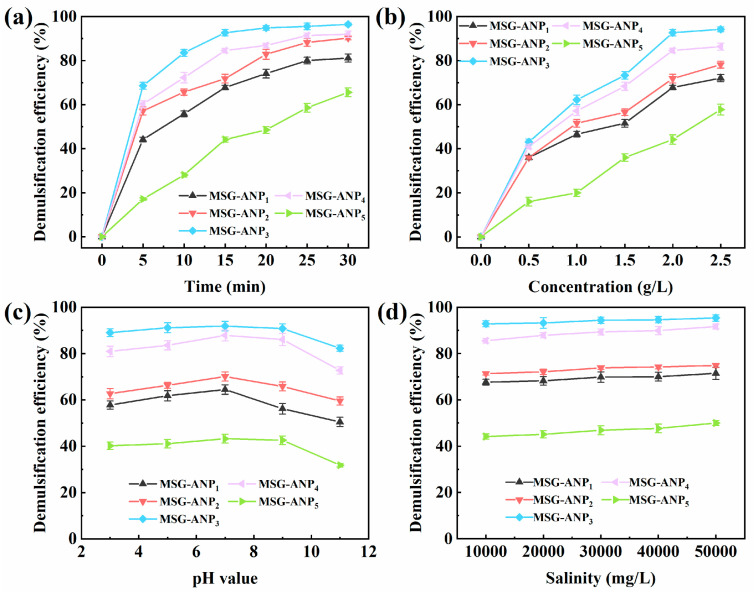
Demulsification performance of MSG-ANP with various mass ratios of MSG to ANP at ambient temperature (**a**); effect of concentration (**b**), pH (**c**), and salinity (**d**) on the demulsification performance of MSG-ANP with various mass ratios of MSG to ANP.

In this study, we investigated the effects of reaction time, dosage, pH and salinity on the emulsion breaking properties of MSG-ANP with different mass ratios. As illustrated in Figure 5a, MSG-ANP with different mass ratios of MSG to ANP were tested at a dosage of 2 g/L at room temperature. Consistent with its favorable wettability and high interfacial activity from the optimal ANP grafting concentration, MSG-ANP_3_ achieved 84% demulsification efficiency after 10 min of reaction, reaching a peak demulsification efficiency of 93% after 15 min. This finding suggests that MSG-ANP_3_ exhibits excellent demulsification performance within a relatively short time frame (Figure S4). Such outstanding performance can be attributed to the high interfacial activity and favorable wettability of MSG-ANP_3_, which enhance its adsorption capability at the oil–water interface in emulsions. Additionally, the presence of oxygen-containing functional groups in MSG-ANP_3_ facilitates the formation of hydrogen bonds with water molecules, thereby disrupting the interfacial film formed by the original surface-active substances, ultimately leading to the rupture of the oil–water interfacial film [35,49].

Furthermore, the dosage of the demulsifier significantly influenced the demulsification performance of MSG-ANP. As illustrated in Figure 5b, after various dosages of MSG-ANP were reacted at room temperature for 15 min, the demulsification efficiency exhibited an increasing trend with the rising dosage of MSG-ANP. Notably, MSG-ANP_3_ demonstrated the highest demulsification efficiency across all dosages, particularly at 2.5 g/L, where it achieved 95%. Although the demulsification efficiency of MSG-ANP_3_ at 2.5 g/L was marginally higher than that at 2 g/L, this difference was not significant. Therefore, to balance demulsification performance with the dosage of the demulsifier, subsequent tests were conducted using a dosage of 2 g/L. The optimal performance of MSG-ANP_3_ (mass ratios of MSG to ANP: 2:1) arises from balanced interfacial activity. While ANP grafting enhances hydrophilicity through hydrogen bonding and hydration layer formation, excessive ANP in MSG-ANP_4_/_5_ causes steric hindrance limiting access to MOF-74 metal sites (Fe/Co-O) for hydrogen bonding, as observed in HRTEM images, and reduces charge modulation capacity, evidenced by the zeta potential plateau in Figure 4b. TEOS-derived SiO_2_ in MOF-74@SiO_2_ provides hydroxyl groups that improve hydrophilicity, but ANP remains critical for ether chain penetration into surfactant films and alkyl group anchoring at oil–water interfaces [32,34].

The pH of the emulsion significantly influences the stability of emulsions, as well as the structure and amphiphilicity of demulsifiers [52]. MSG-ANP_3_, exhibiting the most favorable balance of properties, demonstrated superior demulsification efficiency under neutral conditions (pH 7.0), achieving optimal performance, with a demulsification rate of 92% within 15 min (Figure 5c and Figure S5). The efficiency remained consistently high under weakly alkaline (pH 9.0) and weakly acidic (pH 5.0) conditions, with marginal reductions of only 1.2% and 0.8%, respectively. However, a significant decline in performance was observed under strongly alkaline conditions (pH 11.0), where the efficiency decreased by 10.4%. In contrast, under strongly acidic conditions (pH 3.0), the efficiency reduction was limited to 3.0%, correlating well with its zeta potential behavior, which shows it remains less negatively charged or even positively charged in acid, demonstrating remarkable stability across a wide pH range. This decline in performance may be attributed to the positive charge of MSG-ANP_3_ in acidic environments, which generates electrostatic repulsion with similarly charged oil droplets, thereby enhancing the stability of the droplets and hindering their aggregation and merging. Conversely, the high ion density in acidic conditions may induce a charge shielding effect, partially mitigating the electrostatic repulsion and resulting in a demulsification efficiency that remains higher than that observed in alkaline environments. Under alkaline conditions, MSG-ANP_3_ acquires a negative charge, which exerts an attractive force on the positively charged surfaces of the oil droplets, partially neutralizing their positive charge. However, this charge neutralization is insufficient to overcome the overall electrostatic stability, leading to the lowest demulsification efficiency [53]. Overall, MSG-ANP_3_ exhibited effective demulsification performance across various pH values.

Salinity is a crucial factor influencing the characteristics of emulsions and indirectly impacts demulsification efficiency [54]. As illustrated in Figure 5d, the influence of salinity on demulsification efficiency was minimal; however, a slight increase in overall efficiency was observed after 15 min with a dosage of 2 g/L of MSG-ANP_3_ at room temperature. This observation may be explained by the presence of salts, which mitigate the electrostatic repulsion between droplets in the emulsion through a charge shielding effect, thereby reducing the oil–water interfacial tension and, consequently, decreasing the stability of the emulsion [55]. MSG-ANP3 performed robustly across the salinity range tested (Figure S6), maintaining its high efficiency. In conclusion, MSG-ANP_3_ demonstrated remarkable demulsification performance across various salinity conditions, which led to the decision to conduct subsequent demulsification experiments utilizing MSG-ANP_3_.

### 3.3. Evaluation of Demulsification Properties of MSG-ANP

**Figure 6:**
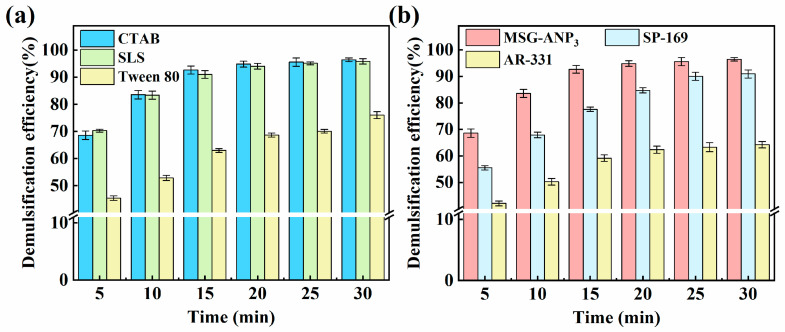
Demulsification performance of MSG-ANP_3_ for different surfactant-stabilized emulsions (**a**); comparison of demulsification performance between MSG-ANP_3_ and commercial demulsifiers (**b**).

With the optimal molar ratio of emulsion breakers screened in the above studies, this part systematically evaluates the emulsion breaking performance of MSG-ANP_3_ in emulsions stabilized by cationic CTAB, anionic SLS, and nonionic Tween 80 surfactants. In addition, the performance of MSG-ANP_3_ is compared with other commercial emulsion breakers. Figure 6a and Figure S7a demonstrate that MSG-ANP_3_ achieved the highest demulsification efficiency of 97% for the CTAB-stabilized emulsion after 30 min. This high efficiency can be attributed to the adsorption of cetyltrimethylammonium ions, generated by the ionization of CTAB, onto the surfaces of the oil droplets. This adsorption increases the positive charge density on these surfaces, which typically enhances the electrostatic repulsion between droplets, thereby stabilizing the emulsion. However, an excessive concentration of positive charges on the surfaces of the oil droplets may conversely diminish electrostatic repulsion, leading to droplet aggregation and subsequent demulsification [56]. For the SLS-stabilized emulsion, the demulsification efficiency was slightly lower but remained high at 96%. The generation of negatively charged dodecyl sulfate ions from the ionization of SLS allows the positively charged MSG-ANP_3_ to neutralize some of the positively charged counter ions in the diffusion layer surrounding the oil droplets. This neutralization reduces the inter-droplet electrostatic repulsion, thus facilitating a higher demulsification efficiency [57]. In contrast, the emulsion stabilized by Tween 80 exhibited a suboptimal demulsification effect, achieving an efficiency of only 76% after 30 min of reaction. This lower efficiency may be attributed to the longer carbon chain of the lipophilic terminal group of the Tween 80 molecule, which contains 18 carbon atoms, compared to 16 in CTAB and 12 in SLS. The extended carbon chain leads to the formation of a thicker monomolecular film of surfactant molecules, which increases the resistance to the aggregation of oil droplets [56].

Figure 6b and Figure S7b illustrate the demulsification efficiency of MSG-ANP_3_ compared to two commercial demulsifiers. The results show that MSG-ANP_3_ achieves a demulsification efficiency of 96% within 30 min, significantly outperforming AR-331 at 64% and slightly exceeding SP-169 at 91%. Notably, MSG-ANP_3_ reaches 93% efficiency in just 15 min, surpassing SP-169, which achieves 78% within the same time frame. These findings indicate that MSG-ANP_3_ not only excels in overall demulsification efficiency but also provides effective results in a relatively short time, suggesting its distinct advantages for rapid demulsification and indicating promising potential for practical applications.

### 3.4. Demulsification Mechanism

To further elucidate the demulsification mechanism of MSG-ANP_3_, this study comparatively analyzed the demulsification efficiencies of MS, MSG, and MSG-ANP_3_. The analysis was conducted after reacting 2 g/L of each material with oil-in-water emulsions for 30 min at room temperature, as shown in Figure S8a. The introduction of the silane coupling agent GPTMS into MS resulted in an increase in demulsification efficiency from 33% to 64%. This enhancement is primarily attributed to the additional oxygen-containing groups (e.g., silyloxy and ester groups) introduced by GPTMS, which facilitate the formation of hydrogen bonds with water molecules. Consequently, this improvement enhances the adsorption capacity of the demulsifier at the oil–water interface [35,58]. Furthermore, grafting ANP and optimizing the mass ratios of MSG to ANP to improve wettability led to the development of MSG-ANP_3_, which demonstrated a demulsification efficiency of up to 93% after 15 min of reaction. This significant improvement can be attributed to the increased content of oxygenated groups (e.g., hydroxyl groups and ether bonds) in the demulsifier, as well as the amphiphilic nature of the grafted ANP. Specifically, the increased content of oxygenated groups enhances the hydrogen bonding with water molecules, while the amphiphilic nature of the grafted ANP facilitates the dispersion of demulsifier molecules at the oil–water interface. These features effectively disrupt the bonds between the original surface-active substances and the oil–water interface, leading to the rupture of the interfacial film [50]. In addition, MSG-ANP_3_ exhibits a low positive charge, enabling it to electrostatically interact with the positively charged counterions in the diffusion layer surrounding the oil droplets. This electrostatic interaction, mediated by gravitational forces, reduces the repulsive forces between the oil droplets, thereby contributing to the destabilization of the emulsion [59].

IFT is a critical indicator of the adsorption capacity of demulsifier molecules at the oil–water interface, playing a vital role in assessing their interfacial activity [51]. As shown in Figure S8b, the initial IFT of pure water and diesel fuel was measured at 39.12 mN/m, with a slight decrease as time progresses. In contrast, the introduction of the demulsifier led to a significant reduction in IFT, indicating the adsorption of demulsifier molecules at the oil–water interface. Notably, the IFT of MSG-ANP_3_, following the grafting of ANP, decreased to 18.17 mN/m at 1200 s, which is significantly lower than the IFT values of MS (28.08 mN/m) and MSG (26.37 mN/m). This observation suggests that the chemical modification through ANP grafting effectively enhances the capability of the demulsifier to reduce interfacial tension and improves its interfacial activity.

The dynamic IFT for varying concentrations of MSG-ANP_3_ is depicted in Figure S8c, revealing that dynamic IFT decreases with increasing concentrations of the demulsifier over time. This phenomenon can be categorized into three distinct stages: (i) rapid diffusion of MSG-ANP_3_ to the oil–water interface, resulting in a sharp decline in IFT; (ii) deceleration in the rate of IFT reduction due to spatial site resistance as the amount of adsorbed MSG-ANP_3_ increases; (iii) equilibrium adsorption of MSG-ANP_3_ at the oil–water interface, leading to the stabilization of IFT [60].

**Figure 7:**
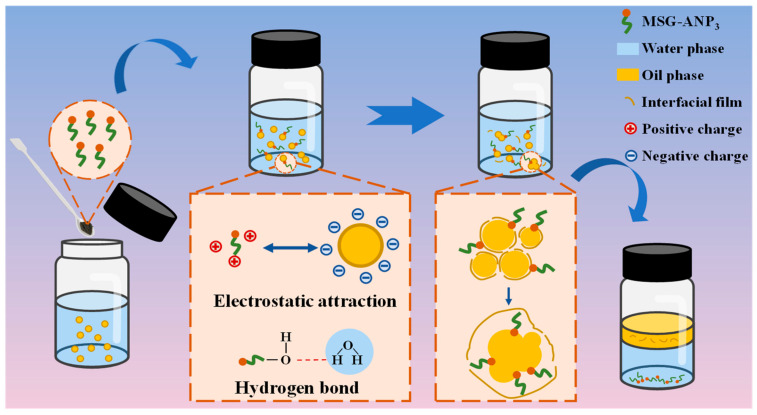
Schematic illustration of the demulsification mechanism of MSG-ANP_3_ for oil-in-water emulsions.

Based on the experimental results of this work, a potential demulsification mechanism has been proposed, as illustrated in Figure 7. MSG-ANP_3_, owing to its excellent amphiphilicity and high interfacial activity, rapidly diffuses to the oil–water interface upon introduction into oil-in-water emulsions. This process involves two primary aspects. Initially, the Fe-O and Co-O metal-oxygen groups [58], along with other oxygen-containing groups such as silyloxy, carbonyl, ether, and hydroxyl groups [35], within the MSG-ANP_3_ molecule can form hydrogen bonds with water molecules. These interactions enhance the adsorption capacity of MSG-ANP_3_ at the oil–water interface, thereby strengthening its interaction with water droplets and creating favorable conditions for the rupture of the interfacial film. Subsequently, the incorporation of MSG-ANP_3_ neutralizes the negative surface charge of the oil droplets in the oil-in-water emulsion, which diminishes the electrostatic repulsion between the droplets. This reduction in repulsion decreases the distance between the oil droplets, thereby enhancing the van der Waals forces of attraction and facilitating their aggregation. Furthermore, MSG-ANP_3_ adsorbs onto the oil–water interface through electrostatic attraction and replaces the original interfacial active substances, resulting in a significant reduction of IFT at the oil–water interface. This decrease in interfacial tension destabilizes the interfacial film, thereby accelerating the demulsification process. Consistent with the discussion in Section 3.2, the medium alkyl chains (C_12_–C_14_) of ANP facilitate deeper penetration at the interface compared to the short silanol groups derived from TEOS, as demonstrated in Figure 7. This directly elucidates the enhanced demulsification performance of MSG-ANP_3_ relative to its silica-modified MOF-74.

## 4. Conclusions

This study successfully synthesized MSG-ANP_3_, a demulsifier derived from MS modified with the silane coupling agent GPTMS and grafted with the nonionic polyether ANP. The demulsification performance of MSG-ANP_3_ within an O/W emulsion system was systematically evaluated. The optimal mass ratio of MSG to ANP was determined to be 2:1, corresponding to MSG-ANP_3_, by assessing demulsification efficiency under varying conditions, including time, demulsifier dosage, pH, and salinity. MSG-ANP_3_ demonstrated exceptional demulsification capability for oil-in-water emulsions at ambient temperature, achieving a demulsification efficiency of 93% at a concentration of 2 g/L within just 15 min. Furthermore, MSG-ANP_3_ maintained excellent demulsification performance across a wide pH range (3.0–11.0) and in high salinity environments (up to 50,000 mg/L). Notably, a demulsification efficiency of 89% was recorded under acidic conditions at pH 3.0 after 15 min at room temperature, significantly expanding its potential applications. The underlying mechanism of demulsification involves electrostatic interactions and hydrogen bonding with MSG-ANP_3_, with electrostatic interactions serving as the primary driving force for enhanced demulsification efficiency. Overall, MSG-ANP_3_ demonstrates promising application prospects and considerable practical value as a demulsifier capable of rapidly and effectively treating oil-in-water emulsions across a broad range of pH levels and high salinity conditions at room temperature.

## Data Availability

The original contributions presented in this study are included in the article/Supplementary Material. Further inquiries can be directed to the corresponding authors.

## Supplementary Materials

The following supporting information can be downloaded at: https://www.mdpi.com/article/10.3390/polym17172386/s1, Text S1. Calculation of grafted ANP molar content from TGA data. Figure S1. Microscopic images of CTAB-emulsions on the day of preparation (a) and after two weeks (b). Figure S2. HRTEM image and elemental line scan spectra of Fe, Co, Si, C and O for MSG-ANP. Figure S3. High-resolution C 1s XPS spectra (a), Fe 2p XPS spectra (b), and Co 2p XPS spectra (c) of MSG-ANP1, MSG-ANP3 and MSG-ANP5. Figure S4. Demulsification performance of MS, MSG, and MSG-ANP after 15 min of reaction at room temperature. Figure S5. Demulsification performance of MSG-ANP3 after 15 min of reaction at room temperature under different pH conditions. Figure S6. Demulsification performance of MSG-ANP3 after 15 min of reaction at room temperature under different salinity conditions. Figure S7. Demulsification performance of MSG-ANP3 after 15 min of reaction at room temperature under different emulsion conditions (a); demulsification performance of commercial demulsifiers (b). Figure S8 Demulsification performance of MS, MSG, and MSG-ANP3 at room temperature (a); IFT (b); IFT of different concentrations of MSG-ANP3 (c). Table S1. Molar content of MSG-ANP. Table S2. Relative atomic content in MSG-ANP.

## References

[1] Ismail N.H., Salleh W.N.W., Ismail A.F., Hasbullah H., Yusof N., Aziz F., Jaafar J. (2020). Hydrophilic polymer-based membrane for oily wastewater treatment: A review. Sep. Purif. Technol..

[2] Gu J., Ji L., Xiao P., Zhang C., Li J., Yan L., Chen T. (2021). Recent progress in superhydrophilic carbon-based composite membranes for oil/water emulsion separation. ACS Appl. Mater. Interfaces.

[3] Husain A., Al-Harthi M.A. (2023). Chemical treatment of oilfield wastewater and the effect of temperature on treatment efficiency: A review. J. Pet. Sci. Eng..

[4] Grenoble Z., Trabelsi S. (2018). Mechanisms, performance optimization and new developments in demulsification processes for oil and gas applications. Adv. Colloid Interface Sci..

[5] Ali N., Bilal M., Khan A., Ali F., Yang Y., Khan M., Adil S.F., Iqbal H.M.N. (2020). Dynamics of oil-water interface demulsification using multifunctional magnetic hybrid and assembly materials. J. Mol. Liq..

[6] ZHOU T., GAO T., LIU C. (2024). Study on the effect of temperature on deposition of virus fine particles by thermophoresis. J. Inn. Mong. Univ. Technol. (Nat. Sci. Ed.).

[7] Faisal W., Almomani F. (2022). A critical review of the development and demulsification processes applied for oil recovery from oil in water emulsions. Chemosphere.

[8] Jamaly S., Giwa A., Hasan S.W. (2015). Recent improvements in oily wastewater treatment: Progress, challenges, and future opportunities. J. Environ. Sci..

[9] Zolfaghari R., Fakhru’l-Razi A., Abdullah L.C., Elnashaie S.S.E.H., Pendashteh A. (2016). Demulsification techniques of water-in-oil and oil-in-water emulsions in petroleum industry. Sep. Purif. Technol..

[10] Shehzad F., Hussein I.A., Kamal M.S., Ahmad W., Sultan A.S., Nasser M.S. (2018). Polymeric surfactants and emerging alternatives used in the demulsification of produced water: A review. Polym. Rev..

[11] Raffa P., Wever D.A.Z., Picchioni F., Broekhuis A.A. (2015). Polymeric surfactants: Synthesis, properties, and links to applications. Chem. Rev..

[12] Qi J., Liang D., Sun H. (2024). Adsorption of Co atom on NC support and reaction mechanism of benzyl alcohol oxidation. J. Inn. Mong. Univ. Technol. (Nat. Sci. Ed.).

[13] Guo K., Li H., Yu Z. (2016). In-situ heavy and extra-heavy oil recovery: A review. Fuel.

[14] Wang D., Yang D., Huang C., Huang Y., Yang D., Zhang H., Liu Q., Tang T., Gamal El-Din M., Kemppi T. (2021). Stabilization mechanism and chemical demulsification of water-in-oil and oil-in-water emulsions in petroleum industry: A review. Fuel.

[15] Wang N., Fuh J.Y.H., Dheen S.T., Senthil Kumar A. (2021). Synthesis methods of functionalized nanoparticles: A review. Bio-Des. Manuf..

[16] Elmobarak W.F., Almomani F. (2021). Functionalization of silica-coated magnetic nanoparticles as powerful demulsifier to recover oil from oil-in-water emulsion. Chemosphere.

[17] Ye F., Wang G., Ao Y., Shen L., Yang Y., Feng X., Zhang Z., Yuan H., Mi Y., Yan X. (2022). Recyclable amine-functionalized carbon nanotubes for the separation of oily wastewater. Chemosphere.

[18] Zhou J., Sui H., Ma J., Li X., Al-Shiaani N.H., He L. (2021). Fast demulsification of oil-water emulsions at room temperature by functionalized magnetic nanoparticles. Sep. Purif. Technol..

[19] Liu C., Wei L., Zhang L., Li Z., Jia X., Geng X. (2022). Preparation of Carbon-Based Nanodemulsifiers Derived from ZIF-8 and their Demulsification Performance for Water-in-Oil Emulsions. ChemistrySelect.

[20] Dhaka S., Kumar R., Deep A., Kurade M.B., Ji S.-W., Jeon B.-H. (2019). Metal–organic frameworks (MOFs) for the removal of emerging contaminants from aquatic environments. Coord. Chem. Rev..

[21] Wu M., Zhai M., Li X. (2021). Adsorptive removal of oil drops from ASP flooding-produced water by polyether polysiloxane-grafted ZIF-8. Powder Technol..

[22] Lin K.-Y.A., Chen Y.-C., Phattarapattamawong S. (2016). Efficient demulsification of oil-in-water emulsions using a zeolitic imidazolate framework: Adsorptive removal of oil droplets from water. J. Colloid Interface Sci..

[23] Leng C., Xu X., Xu M., Guo N., Ai L., Dai J., Feng S., Zhang S., Wang L. (2025). Enhancing demulsification of carbon fiber composite membranes via the controllable charge effect for saline emulsion separation. Sep. Purif. Technol..

[24] Meng J., Teng J., Li F., Li T., Greco R., Cao W. (2025). Sm-MOF decorated cotton for efficient on-demand oil-water separation and organic pollutants removal. Sep. Purif. Technol..

[25] Xue J., Lv Y., Zhang J., Huang K., Qu J., Wang M., Ma S. (2025). UiO-66-NH_2_/hydrothermal carbon nanocomposite assembled mesh membrane with photocatalytic self-cleaning and antibacterial properties for on-demand oily wastewater separation. Sep. Purif. Technol..

[26] Chen Z., Xue J., Zhang J., Qu J., Huang K., Wang M. (2024). Co-doped Zr-UiO-66-NH2@carboxylated cellulose nanocrystals/PAN membrane for oil/water separation with photocatalysis-PMS synergistic self-cleaning and antibacterial activity. Int. J. Biol. Macromol..

[27] Awwad M., Bilal M., Sajid M., Nawaz M.S., Ihsanullah I. (2023). MOF-based membranes for oil/water separation: Status, challenges, and prospects. J. Environ. Chem. Eng..

[28] Chi H., Wan J., Ma Y., Wang Y., Ding S., Li X. (2019). Ferrous metal-organic frameworks with stronger coordinatively unsaturated metal sites for persulfate activation to effectively degrade dibutyl phthalate in wastewater. J. Hazard. Mater..

[29] Burtch N.C., Jasuja H., Walton K.S. (2014). Water stability and adsorption in metal–organic frameworks. Chem. Rev..

[30] Kalaj M., Bentz K.C., Ayala S., Palomba J.M., Barcus K.S., Katayama Y., Cohen S.M. (2020). MOF-polymer hybrid materials: From simple composites to tailored architectures. Chem. Rev..

[31] Lü T., Zhang X., Ma R., Qi D., Sun Y., Zhang D., Huang J., Zhao H. (2023). Quaternary ammonium siloxane-decorated magnetic nanoparticles for emulsified oil-water separation. Sep. Purif. Technol..

[32] He C., Zhang X., He L., Sui H., Li X. (2022). Revealing the non-covalent interactions between oxygen-containing demulsifiers and interfacially active asphaltenes: A multi-level computational simulation. Fuel.

[33] Zhang X., He C., Zhou J., Tian Y., He L., Sui H., Li X. (2023). Demulsification of water-in-heavy oil emulsions by oxygen-enriched non-ionic demulsifier: Synthesis, characterization and mechanisms. Fuel.

[34] Ding S., Wan J., Ma Y., Wang Y., Pu M., Li X., Sun J. (2021). Water stable SiO_2_-coated Fe-MOF-74 for aqueous dimethyl phthalate degradation in PS activated medium. J. Hazard. Mater..

[35] Zhou J., Zhang X., He L., Sui H., Li X. (2022). Nano-modification of carboxylated polyether for enhanced room temperature demulsification of oil-water emulsions: Synthesis, performance and mechanisms. J. Hazard. Mater..

[36] Xu H., Wang J., Yang X., Ning L. (2021). Magnetically recyclable graphene oxide demulsifier adapting wide pH conditions on detachment of oil in the crude oil-in-water emulsion. ACS Appl. Mater. Interfaces.

[37] Lee B., Moon D., Park J. (2020). Microscopic and mesoscopic dual postsynthetic modifications of metal–organic frameworks. Angew. Chem. Int. Ed..

[38] Wang Q., Lu J., Jiang Y., Yang S., Yang Y., Wang Z. (2022). FeCo bimetallic metal organic framework nanosheets as peroxymonosulfate activator for selective oxidation of organic pollutants. Chem. Eng. J..

[39] Ding S., Wan J., Wang Y., Yan Z., Ma Y. (2021). Activation of persulfate by molecularly imprinted Fe-MOF-74@SiO2 for the targeted degradation of dimethyl phthalate: Effects of operating parameters and chlorine. Chem. Eng. J..

[40] Ma J., Yang Y., Li X., Sui H., He L. (2021). Mechanisms on the stability and instability of water-in-oil emulsion stabilized by interfacially active asphaltenes: Role of hydrogen bonding reconstructing. Fuel.

[41] Tóth A., Veres M., Kereszturi K., Mohai M., Bertóti I., Szépvölgyi J. (2011). Structure–property and composition–property relationships for poly(ethylene terephthalate) surfaces modified by helium plasma-based ion implantation. Appl. Surf. Sci..

[42] Xu Y., Wang G., Zhu L., Deng W., Wang C., Ren T., Zhu B., Zeng Z. (2022). Desert beetle-like microstructures bridged by magnetic Fe_3_O_4_ grains for enhancing oil-in-water emulsion separation performance and solar-assisted recyclability of graphene oxide. Chem. Eng. J..

[43] Lu X., Gu L., Wang J., Wu J., Liao P., Li G. (2017). Bimetal-organic framework derived CoFe_2_ O_4_ /C porous hybrid nanorod arrays as High-performance electrocatalysts for oxygen evolution reaction. Adv. Mater..

[44] Lü T., Zhang S., Qi D., Zhang D., Vance G.F., Zhao H. (2017). Synthesis of pH-sensitive and recyclable magnetic nanoparticles for efficient separation of emulsified oil from aqueous environments. Appl. Surf. Sci..

[45] Ye F., Jiang X., Liu H., Ai G., Shen L., Yang Y., Feng X., Yuan H., Zhang Z., Mi Y. (2022). Amine functional cellulose derived from wastepaper toward oily wastewater treatment and its demulsification mechanism. J. Mol. Liq..

[46] Shen L., Ai G., Liu H., Zhu L., Lai L., Yan X., Yu W., Mi Y. (2024). Synthesis and demulsification performance of a novel low-temperature demulsifier based on trimethyl citrate. J. Hazard. Mater..

[47] Shen X., Wang J., Xin G. (2021). Effect of the zeta potential on the corrosion resistance of electroless nickel and PVDF composite layers using surfactants. ACS Omega.

[48] Li Z., Chakraborty A., Fuentes J., Zamora E., Vázquez F., Xu Z., Liu Q., Flores C., McCaffrey W.C. (2021). Study on demulsifier crude oil interactions at oil-water interface for crude oil dehydration. Colloids Surf. A Physicochem. Eng. Asp..

[49] Tian Y., Qi Y., Chen S., Han H., Wang H., Gong X., Zhang M., Jiang X., Chen Y. (2023). Recombination of hydrogen bonds clipping interfacial film effectively for dehydrated tight oil. Sep. Purif. Technol..

[50] Yan S., Jiang P., Zhang X., Guo Y., Fang W. (2023). Cryogenic efficient phase separation of oil–water emulsions with amphiphilic hyperbranched poly(amido-amine). J. Mater. Chem. A.

[51] Zhang L., Wei L., Shi L., Dai X., Guo S., Jia X., Liu C. (2022). Synthesis and characterization of a novel reticulated multi-branched fluorinated polyether demulsifier for w/o emulsion demulsification. J. Polym. Res..

[52] Hao X., Elakneswaran Y., Afrin S., Shimokawara M., Kato Y., Kitamura R., Hiroyoshi N. (2023). Role of pH and cations on emulsion formation and stability of crude oils. Geoenergy Sci. Eng..

[53] Shen L., Liu T., Li H., Li S., Hu Z., Jiang X., Liu H., Zhang Z., Mi Y., Yu W. (2024). Quadruple-branched jellyfish-like demulsifier used for completely demulsifying water-in-oil emulsion at low temperature and its demulsification mechanism. Fuel.

[54] Al-Sakkaf M.K., Onaizi S.A. (2023). Crude oil/water nanoemulsions stabilized by rhamnolipid biosurfactant: Effects of acidity/basicity and salinity on emulsion characteristics, stability, and demulsification. Fuel.

[55] Onaizi S.A. (2022). Effect of salinity on the characteristics, pH-triggered demulsification and rheology of crude oil/water nanoemulsions. Sep. Purif. Technol..

[56] Faizullayev S., Adilbekova A., Kujawski W., Mirzaeian M. (2022). Recent demulsification methods of crude oil emulsions—Brief review. J. Pet. Sci. Eng..

[57] Björkegren S., Nordstierna L., Törncrona A., Palmqvist A. (2017). Hydrophilic and hydrophobic modifications of colloidal silica particles for Pickering emulsions. J. Colloid Interface Sci..

[58] Lin L., Zhang C., Liang C., Zhang H., Wang Z., Wang P., Zheng Z., Cheng H., Xing D., Dai Y. (2024). Hydrogen bonds induced ultralong stability of conductive π–d conjugated FeCo_3_ (DDA)_2_ with high OER activity. Adv. Mater..

[59] Fan Y., Kellermeier M., Xu A.Y., Boyko V., Mirtschin S., Dubin P.L. (2017). Modulation of Polyelectrolyte–Micelle Interactions via Zeta Potentials. Macromolecules.

[60] Zhou W., Cao X., Guo L., Zhang L., Zhu Y., Zhang L. (2018). Interfacial dilational properties of polyether demulsifiers: Effect of branching. Colloids Surf. A Physicochem. Eng. Asp..

