# Supplementary material for: Acid/Alkali-Resistant Modified MOF-74 Grafted with Polyether Demulsifier for Oil-in-Water Emulsions Under Ambient Conditions
Source: Polymers (Basel). 2025 Aug 31;17(17):2386. doi: 10.3390/polym17172386 (PMC12431512; doi:10.3390/polym17172386)
Supplement: Supplementary file 1 [file polymers-17-02386-s001.zip › polymers-3754338-supplementary.pdf]

Supporting information

Acid/Alkali-Resistant Modified MOF-74 Grafted with Polyether Demulsifier for Oil-in-Water Emulsions under Ambient Conditions

Bingyu Wang[1], Wei Guo[1,*], Ying Deng[1], Wenbin Jiao[1], Linzhu Du[1], Junhui Yue[1], Bo Zhang[2,*]

[1]National Engineering Laboratory for Advanced Municipal Wastewater Treatment and Reuse Technology, Beijing University of Technology, Beijing 100124, China.

[2] Chinese Research Academy of Environmental Sciences (CRAES), Beijing 100012, China.

**Corresponding author: Wei Guo and Bo Zhang*

gwfybj@bjut.edu.cn (Wei Guo); zhangbo@craes.org.cn (Bo Zhang)

Full postal address: Beijing University of Technology, Pingleyuan 100, Chaoyang, Beijing 100124, China.

Text S1 Calculation of grafted ANP molar content from TGA data

The mass of grafted ANP (m_{ANP}) was obtained from the difference in weight loss between MSG-ANP and MSG:

$$mANP = (\triangle \mathrm{w}_{ANP} - \triangle \mathrm{w}_{MSG-ANP}) \times m_0 \qquad \text{(S1)}$$

where Δw_{MSG} and $\Delta w_{MSG\text{-}ANP}$ are the weight-loss percentages (200–800 °C) for MSG and MSG-ANP, respectively, and m_0 (15 mg) is the initial sample mass.

The molar content n_{ANP} (mmol g^{-1}) of grafted ANP (M_{ANP} = 638.49 g mol^{-1}) was then calculated as:

$$n_{ANP} = \frac{m_{ANP}}{M_{ANP}} \times 1000 \qquad \text{(S2)}$$

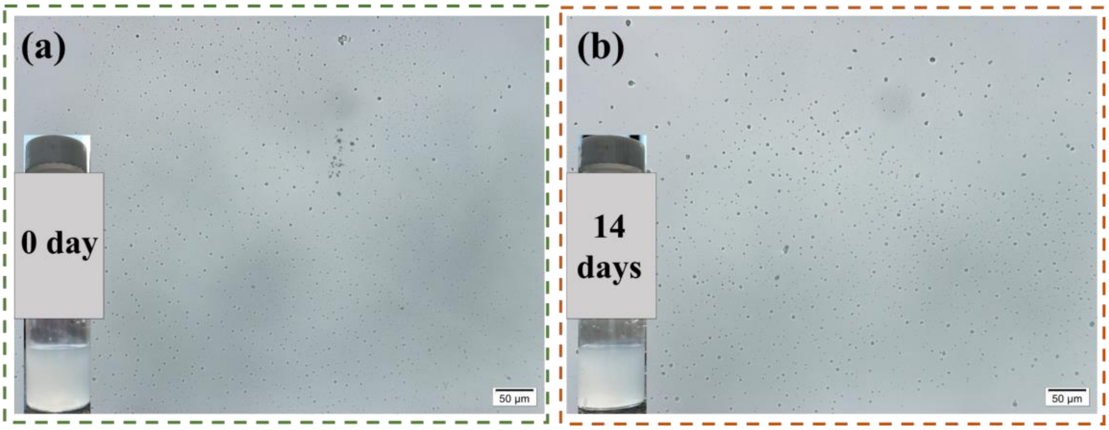

Figure. S1. Microscopic images of CTAB-emulsions on the day of preparation (a) and after two weeks(b).

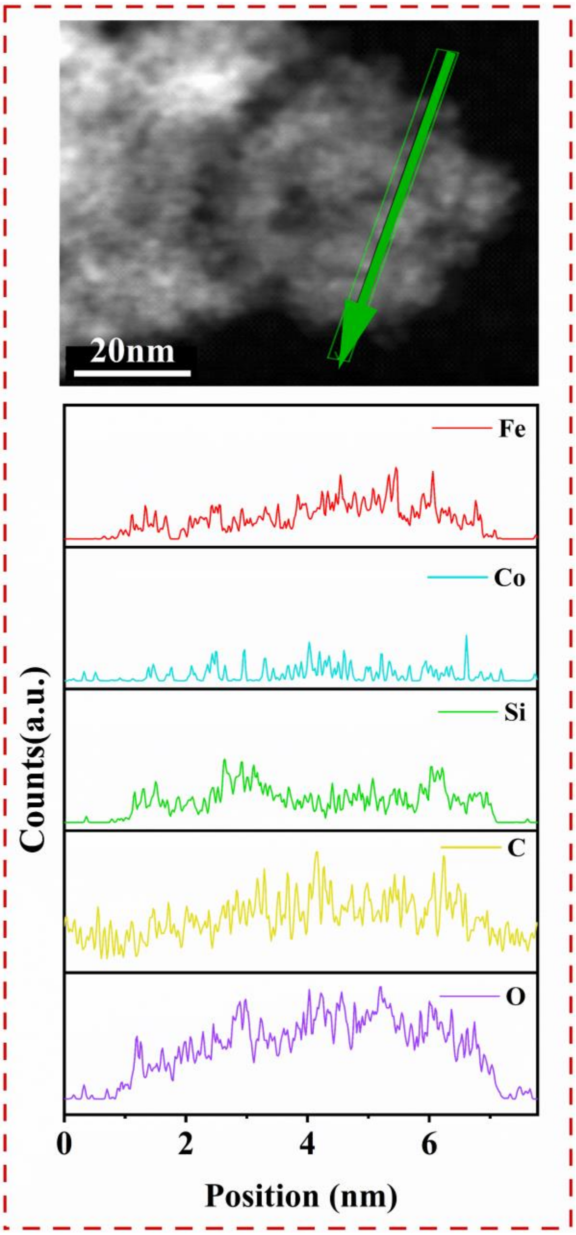

Figure. S2. HRTEM image and elemental line scan spectra of Fe, Co, Si, C and O for MSG-ANP.

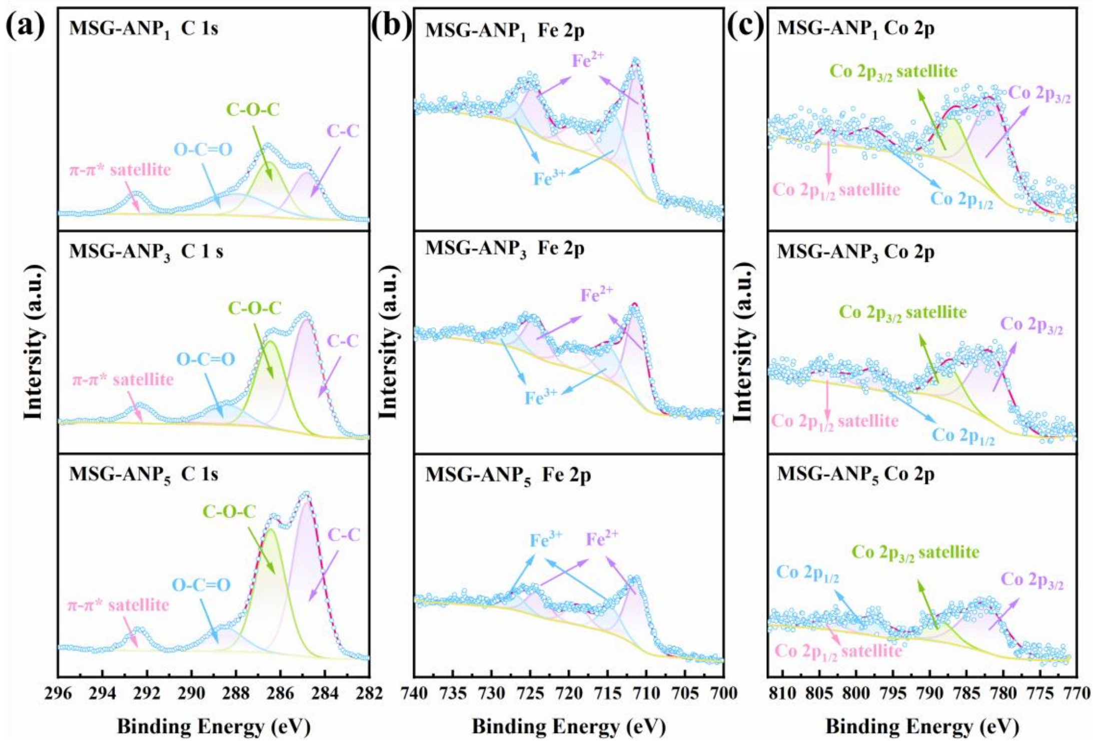

Figure. S3. High-resolution C 1s XPS spectra (a), Fe 2p XPS spectra (b), and Co 2p XPS spectra (c) of MSG-ANP$_1$、MSG-ANP$_3$ and MSG-ANP$_5$.

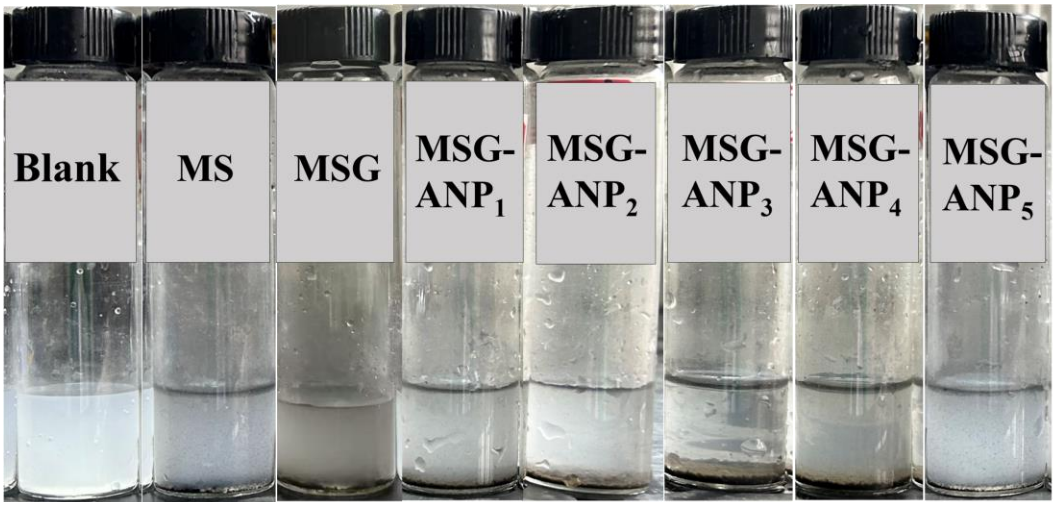

Figure. S4. Demulsification performance of MS, MSG, and MSG-ANP after 15 min of reaction at room temperature.

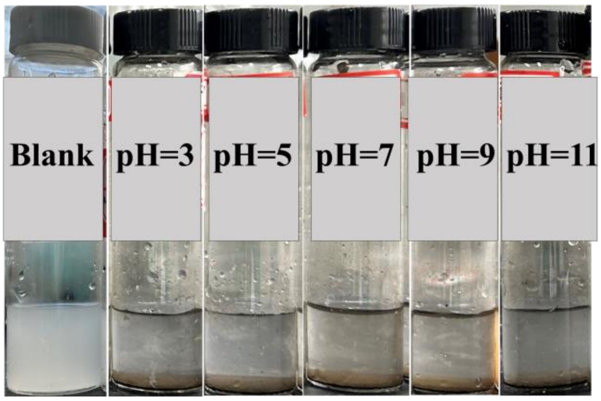

Figure. S5. Demulsification performance of MSG-ANP$_3$ after 15 min of reaction at room temperature under different pH conditions.

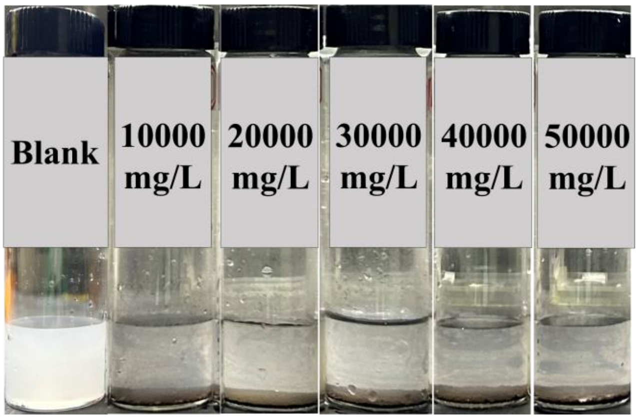

Figure. S6. Demulsification performance of MSG-ANP$_3$ after 15 min of reaction at room temperature under different salinity conditions.

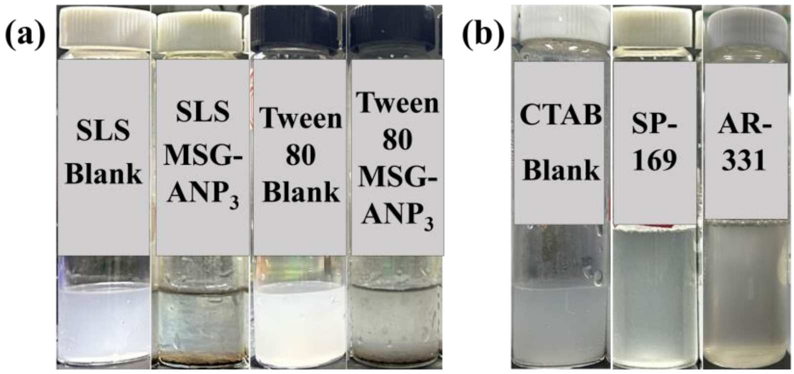

Figure. S7. Demulsification performance of MSG-ANP$_3$ after 15 min of reaction at room temperature under different emulsion conditions (a); demulsification performance of commercial demulsifiers (b).

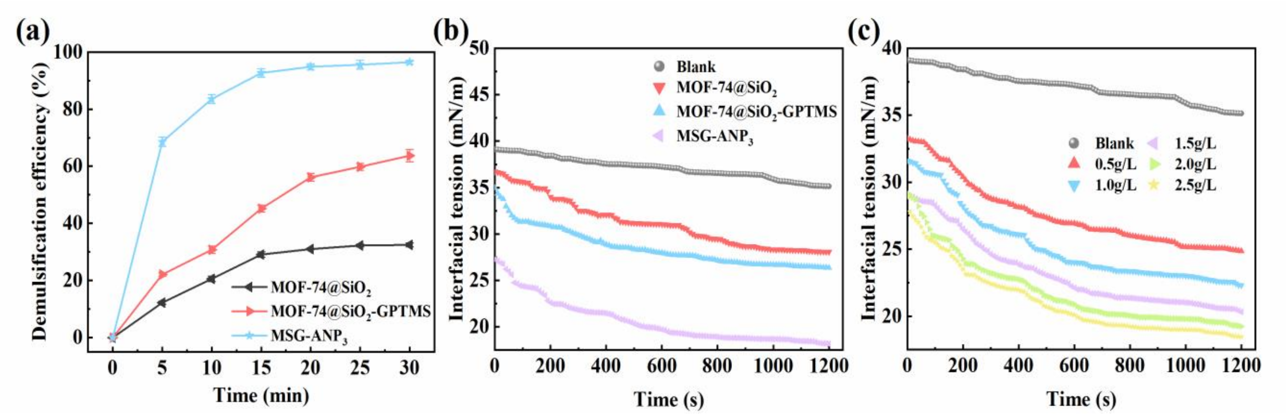

Figure. S8 Demulsification performance of MS, MSG, and MSG-ANP$_3$ at room temperature (a); IFT (b); IFT of different concentrations of MSG-ANP$_3$ (c).

Table S1. Molar content of MSG-ANP.

Samples	Mass ratios of MSG to ANP	The weight loss of the demulsifier at 900°C(wt%)	The molar content of ANP (mmol/g)
MOF-74@SiO_2	-	4.24	0
MOF-74@SiO_2-GPTMS	0	6.18	0
MSG-ANP_1	8:1	9.49	0.08
MSG-ANP_2	4:1	11.36	0.12
MSG-ANP_3	2:1	14.49	0.20
MSG-ANP_4	1:1	17.65	0.27
MSG-ANP_5	1:2	22.09	0.37

Table S2. Relative atomic content in MSG-ANP.

Samples	Relative atom content (%)				
	Fe 2p	Co 2p	Si 2p	C 1s	O 1s
**MOF-74@SiO_2**	7.40	5.04	8.46	27.77	51.33
**MOF-74@SiO_2-GPTMS**	4.78	3.14	7.93	37.01	47.14
**MSG-ANP_1**	3.88	1.97	7.86	33.99	52.30
**MSG-ANP_2**	3.34	1.73	6.84	39.00	49.09
**MSG-ANP_3**	2.34	1.67	5.81	43.36	46.82
**MSG-ANP_4**	1.20	0.62	4.62	49.30	44.26
**MSG-ANP_5**	1.06	0.54	3.40	56.92	38.08
